# Pseudo-HELLP syndrome par carence en folates et/ou en vitamine B12: à propos d'un cas

**DOI:** 10.11604/pamj.2014.18.99.2483

**Published:** 2014-05-27

**Authors:** Mechaal Benali, Mahdi Bouassida, Firas Dhouib, Kaled Bouzeidi

**Affiliations:** 1 Service d'anesthésie réanimation, hôpital Taher Maamouri, Nabeul 8000, Tunisie; 2 Service de chirurgie générale, hôpital Taher Maamouri, Nabeul 8000, Tunisie; 3 Service d'imagerie médicale, hôpital Taher Maamouri, Nabeul 8000, Tunisie

**Keywords:** HELLP syndrome, carence, folates, vitamine B12, HELLP syndrome, deficiency, folates, vitamin B12

## Abstract

Plusieurs pathologies médicales peuvent interférer avec la grossesse et mimer le tableau biologique de HELLP syndrome. L’évolution naturelle de ce syndrome est d'une particulière gravité pour la mère et le fœtus, il convient d’éliminer rapidement les autres diagnostics afin d’éviter une extraction fœtale prématurée injustifiée. Nous rapportons le cas d'une parturiente qui s'est présentée avec un tableau évocateur d'un HELLP syndrome, rapporté finalement à une carence en folates et en vitamine B12.

## Introduction

Le HELLP syndrome est une micro angiopathie thrombotique gravidique, qui associe une hémolyse, une thrombopénie et une cytolyse hépatique. C'est une forme clinique particulière de pré éclampsie et il peut survenir de façon isolée ou incomplète [1]. Cependant plusieurs autres pathologies médicales peuvent interférer avec la grossesse et mimer le tableau biologique de hellp syndrome [2] L’évolution naturelle du HELLP syndrome étant d'une particulière gravité pour la mère et le fœtus[3], il convient d’éliminer rapidement les autres diagnostics afin d’éviter une extraction fœtale prématurée injustifiée. Nous décrivons le cas d'une parturiente qui s'est présentée avec un tableau évocateur d'un HELLP syndrome, rapporté finalement à une carence en folates et/ou en vitamine B12.

## Patient et observation

Il s'agit d'une femme âgée de 39 ans, deuxième geste, deuxième pare, 01enfant vivant, sans antécédent personnel ou familial particulier, qui s'est présentée aux urgences à 32 semaines d'aménorrhée, pour céphalée et douleur abdominal dans un contexte d'altération de l’état générale (amaigrissement, vomissement et asthénie). L'examen initial a retrouvé des œdèmes des membres inférieurs, avec des chiffres de pression artérielle systolique modérément élevée (150/92 mm Hg) et une protéinurie à deux croix.

A l’échographie obstétricale, un retard de croissance a été mis en évidence sans signes de décollement placentaire.

Le bilan biologique a révélé une anémie sévère avec un taux d′hémoglobine à 4.3 g/dl, une thrombopénie à 52 000 /mm3 avec un taux de prothrombine (TP) à 100%, sans cytolyse hépatique ni cholestase associée. La fonction rénale était par ailleurs normale (créatininémie à 74µmol/l).

Devant ce tableau clinico-biologique, le diagnostic d'une pré éclampsie sévère compliquée d'un HELLP syndrome dissocié a été retenu. La patiente a été mise sous l'association sulfate de magnésium – nicardipine. Une césarienne a été pratiquée sous anesthésie générale après une maturation pulmonaire par corticothérapie et une transfusion de 02 culots globulaire. La patiente a été extubée sur table opératoire puis transférée au service de réanimation pour complément de prise en charge. L'accouchement a donné naissance d'un nouveau né en bonne santé (Le score d'Apgar était 7 à 1 minute et 8 à 5 minutes; le poids de naissance 2 kg 800mg).

Le bilan postopératoire a montré une anémie macrocytaire (un taux d'hémoglobine à 5.1 g/dl avec VGM = 103), une thrombopénie à 67.000/mm3 associé à une leucopénie (2000 /mm3) et un taux de globules rouges à 2.54 G/l. par ailleurs, le bilan hépatique, le taux de prothrombine et la fonction rénale ont été normaux.

Devant cette pancytopénie, nous avons réalisé une ponction médullaire avec un myélogramme qui retrouve une moelle riche, avec mégaloblastose. Le dosage de la vitamine B12 et les folates a montré une concentration sérique de vitamine B12 = 126 µg/l (valeurs normales = 200 à 800 µg/l) et la concentration d'acide folique sérique = 2 µmol/l (valeurs normales = 2,5 à 20 µg/l). Le diagnostic d'une anémie macrocytaire mégaloblastique par carence en Vitamine B12 et/ou en folates a été ainsi retenu. La patiente a été mise sous acide folique 5 mg/jour avec une injection journalière de 1 mg de vitamine B12 en intramusculaire. L’évolution a été marquée par la régression de pancytopénie durant le premier mois du traitement substitutif.

## Discussion

Le HELLP syndrome (hemolysis, elevated liver enzymes and low platelet) a été décrit pour la première fois par Weinstein en 1982. Il s'agit d'une microangiopathie thrombotique gravidique, considérée comme une forme clinique grave de la pré éclampsie [2].

La nature vague et non spécifique des plaintes fonctionnelles, peut rendre le diagnostic d′un HELLP syndrome frustrant pour le clinicien. En fait, l′examen physique peut être strictement normal et le HELLP syndrome peut survenir de manière isolée en l'absence de tout signe de prééclampsie dans 15% des cas [3]. Par conséquent, le diagnostic du HELLP syndrome est fondé sur des évidences biologiques: anémie hémolytique, cytolyse hépatique et thrombocytopénie. Toutefois, l'individualisation de ce syndrome peut être difficile en raison de la présence de formes biologiques incomplètes telles qu′une cytolyse hépatique isolée, une hémolyse avec cytolyse hépatique ou une thrombopénie isolée [1, 3]. Ainsi, nous n′avons pas pu rejeter ou retenir le diagnostic de HELLP syndrome chez notre patiente, même en l′absence de cytolyse hépatique.

Cependant, plusieurs pathologies médicales, dont l′anémie mégaloblastique par carence en folates et/ou vitamine B 12, peuvent donner un tableau biologique voisin de celui du HELLP syndrome, et doivent être évoquées avant de décider une extraction fœtale. Frederici et al. ont rapporté deux cas de déficit en vitamine B12 responsables d'un tableau de pseudo microangiopathie thrombotique avec anémie hémolytique et thrombopénie [4]. Deux autres cas de déficit en folates se traduisant par un tableau simulant un HELLP syndrome, ont été publiés par Gupta [5]. De même, parmi les sept cas de pseudo-HELLP décrits par Chauvet et al, quatre patientes ont été césarisées devant des signes cliniques et biologiques évocateurs de HELLP syndrome, et rapportées finalement à une carence vitaminique [2]. Cependant, plusieurs éléments suggéraient d'emblée qu'il ne pouvait pas s'agir d'un syndrome HELLP: le caractère arégénératif de l'anémie, l'absence de schizocytose et surtout le caractère particulièrement marqué de l'anémie qui était comprise dans les sept cas entre 4,5 et 6,4 g/dl, contrastant avec une tolérance inhabituelle pour une déglobulisation aiguë d'autre part, la cytolyse hépatique était extrêmement mineure (les transaminases ne dépassaient 100 UI/l dans aucun cas) et on pouvait remarquer que seules les ASAT étaient augmentées et non pas les ALAT qui sont plus caractéristiques d'une atteinte hépatique.

Donc même l′association d′une hémolyse, thrombopénie et cytolyse hépatique, ne permet pas de se prononcer avec certitude en faveur d′un HELLP syndrome [6]. Chez notre patiente, le caractère bien toléré d'une anémie sévère supposée être aigue ainsi que l′absence de cytolyse hépatique, étaient contre le diagnostic de HELLP syndrome.

## Conclusion

Une carence sévère en folates et/ou en vitamine B12 pendant la grossesse, peut engendrer un tableau compatible avec le diagnostic de HELLP syndrome. Connaissant la gravité potentielle de ce syndrome pour la mère et pour l'enfant, il faut savoir évoquer les différents diagnostics différentiels, dont celui du déficit en folates et en vitamine B12, afin d'apporter un traitement spécifique et d’éviter une extraction fœtale prématurée injustifiée.
